# Descriptive study of the number and duration of physical restraints placed in patientes admitted to an adolescent psychiatric unit from 2020 to 2023

**DOI:** 10.1192/j.eurpsy.2025.1122

**Published:** 2025-08-26

**Authors:** P. Del Sol, L. Mallol, A. Izquierdo, R. Fernández, M. García Moreno, M. Crespo, M. I. Palanca

**Affiliations:** 1 Hospital Puerta de Hierro, Madrid; 2 Hospital Infanta Cristina, Parla, Spain

## Abstract

**Introduction:**

The management of situations of behavioral dyscontrol is essential in hospitalization units. Strategies such as verbal or pharmacological restraint are the first steps to assist in the emotional regulation of a patient with a potentially escalating state of restlessness. In cases where this fails or is not possible, and there is a risk to the patient or others, physical restraints are a strategy for managing the agitated state. The Adolescent Short Stay Unit at Puerta de Hierro Hospital consists of 11 beds. The age of admission is 12 to 17 years.

**Objectives:**

To present data on the number of physical restraints placed in the Adolescent Brief Hospitalization Unit and the average time of placement from 2020 to 2023. The results from 2024 will be presented in the poster of this abstract.

**Methods:**

Physical restraint data were reviewed through patient records and the physical restraint registry that is part of the unit’s protocol.

**Results:**

During **2020**, physical restraints were placed on 8.7% of the patients admitted that year (21 of 240). In that year, a total of 110 physical restraints were placed for a total of 707.73 hours and an average of 7.06 hours. During the year **2021**, 13.3% required physical restraint (30 of 2236). In that year 89 physical restraints were placed for a total of 470.25 hours and an average of 5.35 hours. In **2022**, 6.4% of the patients admitted required physical restraint (15 of 236), 11 of whom were women. In that year a total of 100 physical restraints were placed with a total of 457 hours and an average of 4.57. It should be noted that that year, of the 100 restraints, 52 were on the same patient, with 19 restraints on the second patient requiring the most restraints. In **2023**, 8.2% of patients required mechanical restraint (19 of 229), 14 of whom were women. A total of 169 restraints were placed for a total of 402 hours and an average time of 2.37 hours. This year, 2023, of the 169 restraints, 106 are on the same patient. From January to August **2024** restraints were applied to 10 patient. A total of 58 restraints were placed, with one patient requiring up to 30 restraints.

It should be noted that the patients who require the most physical restraints are patients with a diagnosis of autism spectrum disorder or patients with intellectual disabilities.

**Conclusions:**

A decrease in the average restraint time has been observed(7.06 to 2.37), which we believe is due to greater training on the part of the nursing team. Patients with Autism Spectrum Disorder and patients with Intellectual Disability are those who have received more physical restraint, suggesting that their management requires a structure and intervention different from those offered by the short hospitalization units.

**Disclosure of Interest:**

None Declared

